# Correlation between Salivary Oxidative Stress Biomarkers and Clinical Severity of Radiation-Induced Oral Mucositis in Head and Neck Cancer Patients

**DOI:** 10.1055/s-0045-1809184

**Published:** 2025-05-27

**Authors:** Nurina Febriyanti Ayuningtyas, Fatimah Fauzi Basalamah, Gisela Lalita Brahmanikanya, Fatma Yasmin Mahdani, Adiastuti Endah Parmadiati, Desiana Radithia, Diah Savitri Ernawati, Madhu Shrestha, Ulinta Purwati Pasaribu, Satutya Wicaksono

**Affiliations:** 1 Department of Oral Medicine, Faculty of Dental Medicine, Universitas Airlangga, Surabaya, Indonesia; 2 Oral Medicine Specialist Study Programme, Faculty of Dental Medicine, Universitas Airlangga, Surabaya, Indonesia; 3 Department of Diagnostic Sciences, College of Dentistry, Texas A&M University, Dallas, Texas, United States; 4 Department of Radiology, Faculty of Medicine, Universitas Airlangga, Surabaya, Indonesia; 5 Medical Division of Radiation Oncology, Dr. Soetomo General Academic Hospital, Surabaya, Indonesia

**Keywords:** glutathione, superoxide dismutase, malondialdehyde, lactate dehydrogenase, oral mucositis, saliva, head and neck cancer, radiotherapy

## Abstract

**Objective:**

Radiation-induced oral mucositis (RIOM) is the most common side effect of radiotherapy (RT) in head and neck cancer (HNC) patients. Oxidative stress plays a major role in the multistep pathogenesis of RIOM. However, the current understanding of the relationship between salivary biomarkers of oxidative stress and the clinical severity of RIOM remains limited. This study aims to analyze the correlation between salivary oxidative stress biomarkers and the clinical severity of RIOM.

**Materials and Methods:**

This cross-sectional study analyzed the levels of salivary oxidative stress biomarkers from 25 HNC patients who underwent RT using enzyme-linked immunosorbent assay and the clinical grades of RIOM in the cohort. The data were then analyzed using the Spearman's correlation statistical test ( *p* -value < 0.05).

**Results:**

The findings demonstrated a significant correlation between salivary glutathione levels ( *r* : –0.396; *p* : 0.050), superoxide dismutase levels ( *r* : –0.447; *p* : 0.025), malondialdehyde levels ( *r* : 0.479; *p* : 0.015), and lactate dehydrogenase levels ( *r* : 0.460; *p* : 0.025) with the clinical severity of RIOM.

**Conclusion:**

The higher salivary oxidative stress correlates with higher severity of RIOM.

## Introduction

Head and neck cancer (HNC) ranks as the seventh most common cancer worldwide, with over 660,000 new cases annually and a nearly 50% mortality rate. 1 Radiation therapy (RT) remains a preferred treatment for HNC patients. During RT, the oral mucosa often falls within the target area, leading to its exposure to irradiation. 2 This exposure can result in radiation-induced oral mucositis (RIOM), a common and debilitating condition affecting more than 90% of HNC patients undergoing RT. 3

RIOM is an inflammatory reaction in the mucosa of the oral cavity caused by exposure to radiation, resulting in swelling, redness, and ulceration. This condition can lead to severe pain, difficulty eating, an increased risk of infection due to open sores, and a significant impact on overall quality of life. 4 5 6 This condition is more likely to happen in patients who receive cumulative radiation doses > 5,000 cGy or concurrent chemoradiation therapy. 7 The pathophysiology of this pathology involves complex biological processes, including direct deoxyribonucleic acid (DNA) damage, oxidative stress, inflammation, and epithelial cell death. 8 In particular, oxidative stress mediated by the accumulation of reactive oxygen species (ROS) has been implicated as the primary mechanism of tissue injury occurring on RIOM. 9

Oxidative stress occurs when free radicals exceed the body's antioxidants, leading to cell damage. Important molecules like DNA, lipids, and proteins are vulnerable to this damage. Ionizing radiation also produces ROS, particularly superoxide radicals, which significantly contribute to radiation cytotoxicity, including RIOM. 9 10 Malondialdehyde (MDA), the final product of lipid peroxidation, is a reliable indicator of oxidative stress. 11 Additionally, antioxidants such as glutathione (GSH) and superoxide dismutase (SOD), along with enzymes like lactate dehydrogenase (LDH), play crucial roles in preventing and counteracting oxidative damage by neutralizing ROS. 12 13

In this study, we aim to evaluate the correlation between the level of oxidative stress biomarkers and the clinical severity of RIOM in patients undergoing radiotherapy for HNC. Understanding this correlation can provide insights into the pathogenesis of mucositis and help develop strategies to prevent or mitigate its impact.

## Materials and Methods

### Study Design and Participants

This cross-sectional study was conducted at Dr. Soetomo General Hospital in Surabaya, Indonesia, from July to October 2023, with ethical clearance number 0705/KEPK/VII/2023. The research subjects were HNC patients who had undergone radiotherapy. The inclusion criteria were as follows: (1) aged 18 to 70 years, (2) diagnosed with HNC, (3) received a minimum radiation dose of 10 Gy, and (4) consented to participate in the study. The exclusion criteria included patients with other systemic diseases (e.g., diabetes mellitus, Sjögren's syndrome, hypertension, etc.), smokers, or those currently consuming any pharmacological agents that may affect saliva production and composition, as well as patients currently undergoing chemotherapy.

### Sample Collection

Saliva samples were collected once from patients during their radiotherapy cycle. The samples were taken in the morning to minimize variability in saliva composition. Patients were instructed to avoid eating, drinking, or brushing their teeth for at least 1 hour before sample collection. Unstimulated saliva was collected by asking patients to drool passively into a sterile container for 5 minutes. The samples were centrifuged at 3,500 revolutions per minute for 5 minutes at room temperature, after which the supernatant was taken and stored at –80°C.

### Biochemical Analysis

This study selected the levels of GSH, SOD, MDA, and LDH in saliva to describe oxidative stress activity. The levels of these markers were measured using enzyme-linked immunosorbent assay (ELISA) kits: Human Glutathione ELISA Kit (BIOENZY, Catalogue No. BZ-08122410-CPEB), Human Superoxide Dismutase ELISA Kit (Catalogue No. BZ-08128190-EB), Human Malondialdehyde ELISA Kit (Catalogue No. BZ-08121731-EB), and Human Lactate Dehydrogenase ELISA Kit (Catalogue No. BZ-08127470-EB). The ELISA procedure was conducted as instructed by the manufacturer, and the optical density value was measured using a microplate reader at 450 nm for 10 minutes.

### Clinical Assessment of Oral Mucositis

Oral medicine specialists conducted a clinical assessment of the oral cavity. The clinician assessed the severity of oral mucositis (OM) using the clinical severity index scoring system released by the World Health Organization, which is based on the subjective and objective findings of the oral medicine specialist. This index ranges from grade 0 (no OM), grade 1 (erythema and soreness), grade 2 (ulcers), grade 3 (ulcers requiring a liquid diet), to grade 4 (ulcers, oral alimentation not possible). 4

### Statistical Analysis

The statistical analysis was performed using SPSS software version 25. The findings of this study were analyzed descriptively and correlatively using the Spearman's correlation test to determine the relationship between variables ( *p* -value < 0.05).

## Results

### Demographic Characteristics

This study included 25 patients with HNC undergoing radiotherapy. The mean age of the patients was 52.3 years, with a range of 32 to 70 years. The gender distribution was 60% male and 40% female. The most common type of cancer was nasopharyngeal carcinoma, accounting for 48% of the cases.

Table 2 presents the correlation between gender and the severity of RIOM. The data is categorized into three severity levels (1, 2, and 3), and the *p* -value for the correlation is also provided.

For males, 7 individuals (38.9%) were classified as having severity level 1, 7 individuals (38.9%) with severity level 2, and 4 individuals (22.2%) with severity level 3.

For females, 3 individuals (42.9%) were classified as having severity level 1, 4 individuals (57.1%) with severity level 2, and no individuals (0.0%) with severity level 3.

The *p* -value of 0.376 indicates no statistically significant correlation between gender and the severity of RIOM ( *p* > 0.05). Statistical tests applied include the Kolmogorov–Smirnov test for normality and chi-square for the correlation analysis.

### Correlative Analysis of Oxidative Stress and Antioxidant Biomarkers and OM Severity

The descriptive analysis of the levels of oxidative stress biomarkers and the distribution of patients based on the severity of OM is shown in Tables 1 and 2 , respectively. Correlative analysis using Spearman's analysis revealed a significant relationship between the oxidative stress biomarkers and the severity of OM ( Table 3 ). The levels of antioxidants GSH and SOD were observed to have a negative relationship with the severity of OM. Conversely, the levels of MDA and LDH positively correlated with the severity of OM. Additionally, the radiotherapy dosage was also observed to positively correlate with the severity of OM ( *p* < 0.05; *r* : 0.882).

**Table 1:** The level of salivary GSH, SOD, MDA, and LDH during radiation therapy cycle (mean ± SD)

Markers	*N*	Mean ± SD (U/L)
GSH	25	1.34 ± 059
SOD	25	1.38 ± 0.70
MDA	25	1.68 ± 0.59
LDH	25	1.49 ± 0.62

Abbreviations: GSH, glutathione; LDH, lactate dehydrogenase; MDA, malondialdehyde; SD, standard deviation; SOD, superoxide dismutase.

**Table 2:** The correlation between genders and the severity of radiation-induced oral mucositis

Characteristics	Severity of radiation-induced oral mucositis a			*p* -Value
	1 b	2 b	3 b	
Genders				
Male	7 (38.9%)	7 (38.9%)	4 (22.2%)	0.376 c
Female	3 (42.9%)	4 (57.1%)	0 (0.0%)	

a One-way analysis of variance.

b Kolmogorov–Smirnov.

c Chi-square, *p* < *α* : 0.05 stated to be significant.

**Table 3:** Distribution of patients based on the severity of oral mucositis

OM severity grades	*n*	%
Grade 0	0	0.0
Grade 1	10	40.0
Grade 2	11	44.0
Grade 3	4	16.0
Grade 4	0	0.0

Abbreviation: OM, oral mucositis.

Table 4 examines the correlation between radiotherapy dose and levels of GSH, SOD, MDA, and LDH in the saliva of HNC patients undergoing radiotherapy, with respect to the severity of RIOM at Dr. Soetomo General Hospital, Surabaya.

**Table 4:** Correlative analysis between the markers and severity grades

Markers	Severity of oral mucositis			Spearman's analysis	
	1	2	3	Significance	*r*
GSH (mean ± SD)	1.52 ± 0.67	1.39 ± 0.51	0.77 ± 0.06	0.050	–0.396
SOD (mean ± SD)	1.65 ± 0.77	1.39 ± 0.59	0.69 ± 0.28	0.025	–0.447
MDA (mean ± SD)	1.48 ± 0.75	1.65 ± 0.36	2.28 ± 0.20	0.015	0.479
LDH (mean ± SD)	1.23 ± 0.73	1.51 ± 0.39	2.07 ± 0.51	0.21	0.460

Abbreviations: GSH, glutathione; LDH, lactate dehydrogenase; MDA, malondialdehyde; SD, standard deviation; SOD, superoxide dismutase.

Radiotherapy dose - GSH: The correlation coefficient ( *r* ) is –0.208 with a *p* -value of 0.319, indicating no significant correlation.

Radiotherapy dose - SOD: The correlation coefficient ( *r* ) is –0.284 with a *p* -value of 0.169, showing no significant correlation.

Radiotherapy dose - MDA: The correlation coefficient ( *r* ) is 0.311 with a *p* -value of 0.130, indicating no significant correlation.

Radiotherapy dose - LDH: The correlation coefficient ( *r* ) is 0.229 with a *p* -value of 0.271, showing no significant correlation.

The analysis used Pearson's correlation for GSH and MDA and Spearman's correlation for SOD and LDH. A *p* -value of < 0.05 is considered statistically significant, but none of the variables showed a significant correlation in this study.

The reason for not conducting a correlation test between the radiotherapy dose and the levels of oxidants and antioxidants is that there is no relationship between the two. The variation in radiotherapy doses is determined based on the considerations of the doctor responsible for the patient's care.

## Discussion

Despite advancements in RT for HNC, OM remains a significant and debilitating side effect that impacts patient's quality of life and treatment outcomes. 14 Current understanding of the relationship between salivary biomarkers of oxidative stress and the clinical severity of OM is limited. 9 Our study demonstrated important findings about the relationship between the biomarkers of oxidative stress activity and the clinical severity of RIOM in HNC patients. This study found a significant association between the increase of salivary MDA and LDH levels and the severity of OM in patients. Moreover, a significant reduction of antioxidant enzymes (i.e., GSH and SOD) was observed along with the OM aggravation. These findings implicate the promising role of oxidative stress biomarkers as a prognostic variable for OM.

Radiotherapy can cause OM by disrupting cell growth and differentiation, causing mucosal epithelial cell death. This disorder occurs due to DNA damage and cell death due to exposure to radiation. Apart from that, there is also an increase in biological activity, which produces various molecules such as proinflammatory cytokines, stress responders, and cell adhesions, which result in more severe damage. The emergence of OM is closely related to the type of cancer treatment the patient is undergoing, such as the type of chemotherapy drug, the dose and frequency of chemotherapy drug administration, the area irradiated, the volume of tissue irradiated, the type of radiation, the fraction dose, and dose of radiotherapy radiation, as well as the administration of radiotherapy. Patient characteristics such as cancer diagnosis, age, and oral hygiene can also influence the occurrence of OM. 15

In the pathogenesis of RIOM, oxidative stress plays a significant role as it initiates oxidative tissue damage and activates inflammatory pathways. 16 MDA is a final product of cell membrane peroxidation, a process where ROS attack polyunsaturated fatty acids in cell membranes. 11 17 18 Our study showed that the aggravation of OM severity followed the elevation of salivary MDA levels. A previous experimental study by Mohammed et al also reported similar results, where MDA levels were highly elevated in the oral mucosa of an OM mouse model. 10 Additionally, a study by Jayachander et al reported significantly higher salivary MDA levels in HNC patients after RT. 19 Furthermore, our study confirmed that higher oxidative stress levels are found in more severe grades of OM, marked by elevated salivary LDH levels. LDH is released into the extracellular space in the event of cellular damage. Thus, this enzyme can be used as an indirect biomarker of oxidative stress. 20 This is consistent with studies by Mohajertehran et al and Gholizadeh et al, which stated that LDH levels in the saliva of HNC patients were significantly higher than in the healthy control group. 21 22 Combined, these findings indicate that oxidative stress significantly contributes to the wider tissue damage in RIOM.

In the event of oxidative stress, the natural defense mechanism of our body counteracts harmful effects by releasing antioxidants. SOD and GSH are among the most crucial antioxidants in the oral cavity, playing a vital role in maintaining redox balance and protecting cells from oxidative damage. 12 This study found that the levels of these antioxidants are negatively correlated with the severity of OM. Similar results were reported by Derındağ et al, which showed that salivary GSH levels in the preradiotherapy group were higher than in the postradiotherapy group. 23 Moreover, other studies have reported lower SOD levels in HNC patients receiving radiotherapy. 13 24 25 Reduced levels of salivary antioxidants may be caused by increased utilization due to elevated ROS levels after radiotherapy, leading to the depletion of antioxidant levels in saliva.

Moreover, our study also demonstrated the relationship between the radiotherapy dosage and the severity of OM in HNC patients undergoing radiotherapy. This aligns with the study by Bell and Kasi (2023), 29 which states that the incidence and severity of mucositis depend on the radiotherapy dosage. Their study reports that patients receiving high-dose chemotherapy have a 76% risk of developing mucositis, and RIOM occurs in 100% of HNC patients receiving high-dose radiotherapy. 6 26

In this study, the average radiotherapy dose (Gy) was 32.24 ± 12.49 Gy with a median of 30 Gy. Literature shows that mucositis is one of the side effects of radiotherapy. Almost all patients who receive radiotherapy treatment with radiation doses above 30 Gy have OM. 27 According to the National Comprehensive Cancer Network Head and Neck Cancer Guidelines, Version 1, 2018, dose fractionation schedule radiation may vary slightly depending on the institutional preferences. Usually, the radiotherapy dosage provides between 2 Gy/fraction every day (Monday–Friday) for 33 to 35 fractions over all areas of severe disease for a total dose of around 70 Gy. 28

The results of this study indicate that oxidative stress plays a crucial role in the pathogenesis of OM in patients undergoing radiotherapy for HNC. The significant decrease in antioxidant levels (GSH and SOD) and the increase in oxidative stress markers (MDA and LDH) during radiotherapy suggest that oxidative damage contributes to the development and severity of OM.

However, a limitation of our study is the inability to recruit patients with grade 0 and grade 4 OM. This may impede validating the biomarkers as predictors of OM progression. Without data from the full spectrum of severity, our analysis may not fully assess the biomarkers' predictive power, potentially affecting the robustness of our conclusions. Future studies should include a more diverse patient population to validate these biomarkers better.

## Conclusion

The higher salivary oxidative stress correlates with higher severity of RIOM. These findings underscore the role of oxidative stress in the pathogenesis of radiation-induced mucositis and suggest potential therapeutic strategies to protect the oral mucosa and improve patient care. Monitoring these biochemical parameters in saliva could be useful in predicting the onset and progression of OM. It may aid in developing preventive and therapeutic strategies to manage this condition in HNC patients.

**Table 5:** The correlation of GSH, SOD, MDA, and LDH levels in saliva in head and neck cancer patients undergoing radiotherapy with the severity radiation-induce of oral mucositis at Dr. Soetomo General Hospital, Surabaya

Variable	Correlation a	
	*r*	*p* -Value
Radiotherapy dose – GSH	–0.208	0.319 b
Radiotherapy dose – SOD	–0.284	0.169 b
Radiotherapy dose – MDA	0.311	0.130 b
Radiotherapy dose – LDH	0.229	0.271 b

Abbreviations: GSH, glutathione; LDH, lactate dehydrogenase; MDA, malondialdehyde; SOD, superoxide dismutase.

a Pearson's correlation.

b Spearman's correlation, *p* < *α* : 0.05 stated to be significant.
